# Laser-Induced Forward Transfer of Pre-Patterned Solder Paste for High-Aspect-Ratio Deposits

**DOI:** 10.3390/ma18225154

**Published:** 2025-11-13

**Authors:** Chaofan Liang, Chongxin Tian, Yanmei Zhang, Xiuli He, Yanhua Bian, Binxin Dong, Gang Yu, Shaoxia Li

**Affiliations:** 1 Institute of Mechanics, Chinese Academy of Sciences, Beijing 100190, China; liangchaofan@imech.ac.cn (C.L.); zhangyanmei201@mails.ucas.ac.cn (Y.Z.); xlhe@imech.ac.cn (X.H.); bianyanhua@imech.ac.cn (Y.B.); dongbinxin@imech.ac.cn (B.D.); gyu@imech.ac.cn (G.Y.); 2 Center of Materials Science and Optoelectronics Engineering, University of Chinese Academy of Sciences, Beijing 100049, China; 3 School of Engineering Science, University of Chinese Academy of Sciences, Beijing 100049, China

**Keywords:** laser-induced forward transfer, stencil print, solder paste, viscoelasticity, aspect ratio

## Abstract

Precision solder deposition for 3D or flexible substrates remains a persistent challenge in electronic packaging. This study introduces a hybrid process that integrates stencil printing with laser-induced forward transfer (LIFT), employing a customized line-scan trajectory to fabricate high-aspect-ratio solder deposits under large-gap, contactless conditions. Solder paste patterns were first printed on a glass carrier and subsequently transferred using pulsed laser scanning, with high-speed imaging employed to resolve the transfer dynamics. Three transfer regimes—stable, unstable, and no transfer—were identified, with the stable regime exhibiting sequential stages governed by vaporization-induced pressure and the viscoelastic response of the solder paste. The initial aspect ratio (AR) was found to critically influence separation behavior, with AR = 0.3 marking the transition between bridging and cantilevered morphologies. Transferred deposits consistently achieved final aspect ratios approaching 0.7; notably, low-AR (<0.15) patterns showed a 2.2-fold height increase. The process maintains a robust energy window (0.937–1.112 J/cm^2^), offering both mechanistic insight into transfer stability and practical guidance for optimizing solder paste deposition in advanced packaging applications.

## 1. Introduction

The increasing demand for miniaturization, high-density functionality, and the industry-wide adoption of three-dimensional (3D) packaging architectures is driving the need for advanced microfabrication processes capable of precise solder joint placement. These bumps serve as the foundational interconnects for a wide array of packaging technologies, including Ball Grid Arrays (BGAs), Flip-Chips, Wafer-Level Chip Scale Packages, and System-in-Package modules [1,2,3,4]. The solder joint remains the fundamental building block for these advanced interconnects, typically operating on the hundred-micron scale [5,6,7,8].

In electronic packaging, the aspect ratio (height to diameter) of solder joints is a key geometric parameter that governs the formation and reliability of these interconnect structures. A higher aspect ratio increases the stand-off height after reflow, thereby reducing thermo-mechanical stresses from the chip–substrate CTE (Coefficient of Thermal Expansion) mismatch and improving solder joints’ fatigue life [9,10,11,12]. In addition, the larger volume of solder joints that accompanies a high aspect ratio reduces current density, enhancing resistance to electromigration [10]. A greater aspect ratio (height to diameter) of solder joints, for a given solder volume, reduces their footprint, facilitating fine-pitch interconnections, lowering bridging risk, and improving impedance control and signal integrity in high-frequency designs [13,14]. While the solder paste transforms from a process-defined shape into a liquid morphology governed by surface tension during reflow, the final aspect ratio of the solder joint remains fundamentally largely determined by the initial deposit’s geometry [15,16,17]. Nevertheless, producing uniform, high-aspect-ratio deposits at the density demanded by next-generation packaging remains a key manufacturing challenge.

Although techniques such as E-jet, jet printing, stencil printing, and LIFT are widely used, each of these methods exhibits significant limitations when it comes to producing solder paste deposits with large H/D ratios. For micro-dispensing and electrohydrodynamic printing (E-jet), as non-contact transfer technologies, the solder droplets are usually with high resolution. But it faces challenges with paste viscosity, droplet coalescence, and volume stability for larger, tall deposits. Jet printing suffers from issues such as spatter and deposit non-uniformity, and its jetting repeatability could degrade over numerous cycles, leading to process instabilities like nozzle clogging [18,19]. Stencil printing encounters limits set by aperture geometry, where a poor area ratio degrades release efficiency. This degradation is a primary source of manufacturing defects, including aperture clogging, solder bridging, and volume inconsistencies that are detrimental to device reliability [20,21,22,23,24]. Moreover, the technique’s inherently planar nature makes it unsuitable for conformal deposition onto topographically complex 3D substrates [25]. Laser-induced forward transfer (LIFT) offers high spatial resolution for deposition but is constrained by several practical trade-offs. The process requires low-viscosity donor films to facilitate the creation of uniform, large-area coatings for clean transfer; however, this directly conflicts with the high viscosity needed to prevent post-transfer slump [26,27,28,29,30]. Furthermore, LIFT exhibits a high sensitivity to its process parameters. For instance, slight deviations in laser fluence, donor-receiver gap, or film uniformity can induce transfer defects such as splashing and satellite droplets, ultimately hindering its ability to reliably fabricate stable, high-aspect-ratio deposits [26,31,32,33,34].

To overcome these limitations, various hybrid laser transfer techniques have been developed. On the receiver side, surface treatments such as roughening [35] or micro-groove fabrication have been applied to confine the material and define the deposit morphology [36,37]. On the donor side, micro-cavity filling, groove structuring, and focused ion beam (FIB) milling have been utilized to pre-pattern the donor layer and improve uniformity [38]. In addition, Dynamic Release Layer-assisted LIFT has been implemented to mitigate laser-induced damage, with Blister-Actuated LIFT using a sacrificial polymer layer to absorb and redistribute laser energy [39]. However, these approaches generally involve complex steps or costly materials, limiting scalability and industrial applicability.

In this work, a hybrid process integrating stencil printing and LIFT is proposed to address the cost and complexity issues of conventional approaches. A method is provided to enable the fabrication of near-spherical solder paste deposits (100–600 µm in diameter, aspect ratio ≈ 0.7) with high transfer efficiency, yielding stable, pre-reflow deposits that are compatible with industrial reflow soldering. This unique morphology resolves the critical trade-off between fine-pitch compatibility and thermo-electrical performance while promoting the ideal structure for enhanced thermo-mechanical reliability, offering a new pathway for next-generation advanced packaging.

## 2. Materials and Methods

### 2.1. Laser-Induced Transfer System

**Figure 1:**
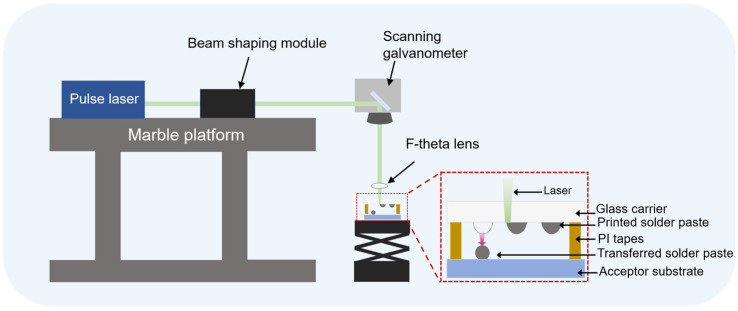
Schematic diagram of the laser-induced transfer system.

The experimental setup for laser-induced transfer of pre-printed solder paste patterns is illustrated in Figure 1. The experimental setup was located in a thousand-level purification standard room. All equipment in the laser system was mounted on a marble base that effectively dampens vibrations to ensure high platform precision. The system employed a pulsed diode-pumped (Advanced Optowave, Inno Laser, Changzhou, China, AMT-532~30 W) laser system, with a wavelength of 532 nm and a pulse width of 12 ps, and the maximum average power was 30 W. The laser beam was guided through an optical path consisting of a beam-shaping system, scanning mirrors, and an F-theta lens. This optical setup delivered a beam spot with a diameter of 300 μm to the interface between the solder paste and the carrier. The beam profile was characterized using a compact beam analyzer (PACON-N0210U, GU OPTICS, Wuhan, China, with built-in absorption attenuator), which confirmed a flat-top beam profile. This profile ensures high energy uniformity within the spot.

**Figure 2:**
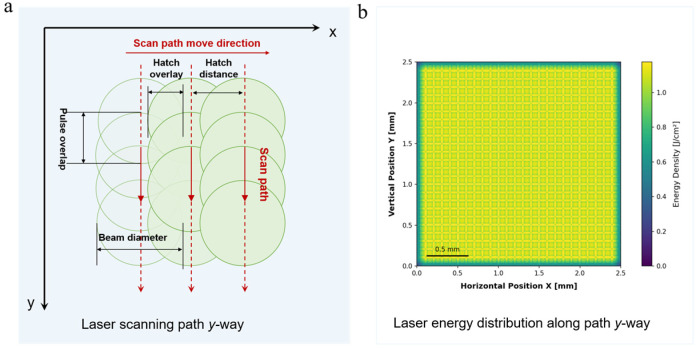
Schematic diagram of the galvanometer scanning path and the corresponding energy distribution. (**a**) Scanning trajectory along the *Y*-axis direction. (**b**) Laser energy distribution within a 50 × 50 spot array region under *Y*-axis scanning.

A high-speed scanning mirror (SCANLAB, Puchheim, Germany, excelli SCAN20) was used, and the focal length of the F-theta lens was 420 mm. In the experiments, the repetition frequency of the scanning mirror was set to 300 kHz, and the scanning speed was 15 m/s. In this work, a beam line-scan method equivalent to laser lift-off (LLO) technology was used to transfer the solder paste pattern, and the path distribution is shown in Figure 2. The hatch overlay was set to 50 μm, and the hatch distance was set to 50 µm, determined by the scanning speed (15 m/s) to frequency (300 kHz).

By computer-controlled laser scanning paths, the printed solder paste pattern could be peeled off and transferred. In this work, a line scanning path was used for the transfer of the solder paste pattern, with the path distribution shown in Figure 2. The pulse repetition frequency along the mirror scanning path direction was set to f = 300 kHz. The energy distribution map in Figure 2b was simulated using a custom Python 3.8 script based on the measured flat-top profile and our scanning parameters. This simulation confirmed a high degree of energy uniformity across the entire scanned area.

### 2.2. Material Selection and Preparation

#### 2.2.1. Materials and Physical Properties

**Figure 3:**
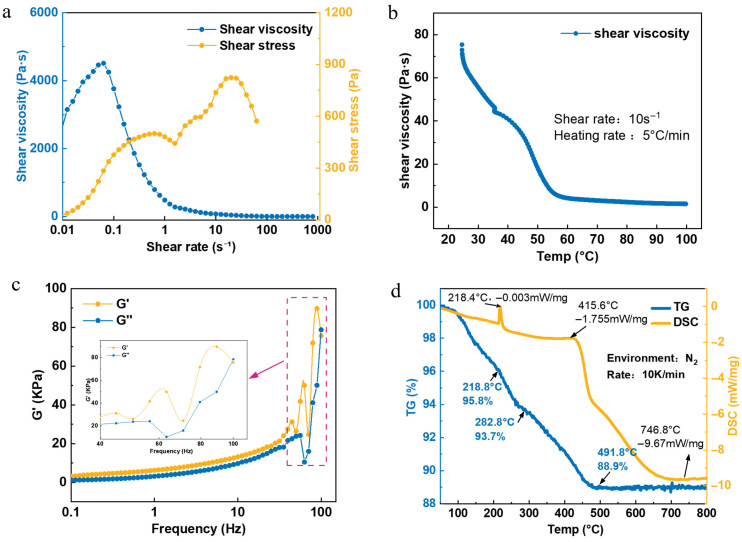
Rheological and thermogravimetric results of the solder paste. (**a**) Viscosity and shear stress vs. shear rate. (**b**) Viscosity vs. temperature. (**c**) Storage (G′) and loss (G″) moduli vs. frequency. (**d**) Thermogravimetric Analysis (TGA) and Differential Scanning Calorimetry (DSC) curves.

Type 6 solder paste (5–15 μm) was selected as it uniquely provides the optimal balance of high-resolution capability and the specific rheological stability required for stable, high-aspect-ratio deposits. The details of the solder paste utilized in this experiment are shown in Table 1. The rheological test results for the Type 6 solder paste selected for the experiment are shown in Figure 3. All tests were conducted using a rotational rheometer in plate mode (NETZSCH Kinexus Prime Lab+, NETZSCH, Selb, Germany).

The yield point occurs at a shear stress of 80~90 Pa, where the storage modulus (G′) equals the loss modulus (G″). Below this stress, elastic behavior dominates (G′ > G″); above it, viscous behavior prevails (G′ < G″) and viscosity declines. Figure 3b shows the variations in G′ and G″ under small-amplitude oscillatory shear at a fixed stress of 5 Pa, over a frequency range of 0.1 to 100 Hz. The G′ and G″ curves intersect at approximately 100 Hz.

#### 2.2.2. Solder Pre-Patterned to Be Transferred

**Figure 4:**
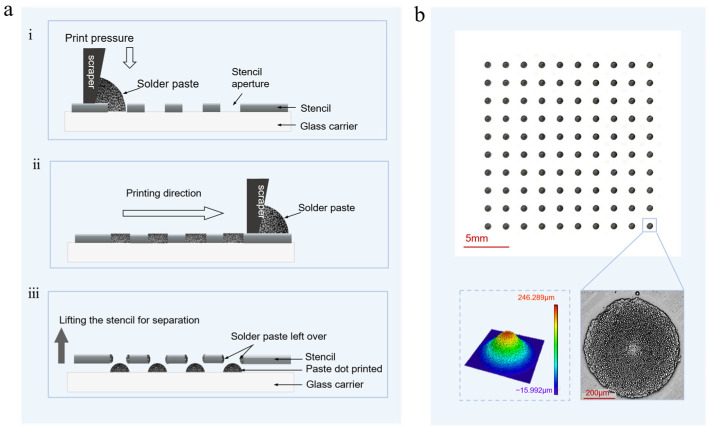
An array of solder paste patterns produced by stencil printing. (**a**) Schematic of the stencil-printing process for solder patterns: (**i**) a squeegee begins to spread the solder paste; (**ii**) the squeegee moves uniformly to fill the stencil apertures. (**iii**) vertical separation of the stencil, leaving the solder paste patterns on the glass carrier. (**b**) Photograph of the array printed on a glass substrate and 2D/3D confocal scans of an individual solder pattern.

As demonstrated in Figure 4, during the printing process of the solder paste pattern, an ultra-thin steel stencil featuring a multi-aperture array was used. The diameter of the aperture was meticulously designed to ensure that the particle size of the filled solder paste (5–15 μm) complies with the Five-Ball Principle [40]. The glass substrate, a rectangular transparent quartz glass plate measuring 186 × 186 mm and 3 mm thick, was selected for the carrier in this experiment. As illustrated in Figure 4(ai), the stencil was initially positioned on the glass carrier. Solder paste was applied uniformly to the non-opening areas of the stencil. Subsequently, as illustrated in Figure 4(aii), a squeegee was gradually maneuvered toward the opening areas of the stencil. This action uniformly filled the solder paste into the stencil apertures along this direction. Subsequently, as illustrated in Figure 4(aiii), the stencil was elevated in a vertical direction. The solder paste retained within the original stencil apertures remained on the glass substrate, forming an array of printed solder paste patterns to be transferred, as depicted in Figure 4b; the 3D and 2D confocal scan images of a single such pattern intended for transfer, which is referred to as the printed solder paste pattern (PSP), are illustrated in Figure 4b.

After the above steps, multiple layers of polyimide tape were adhered to the glass carrier to create a step height, which defines the transfer gap between the carrier and receiver substrates. All printed solder paste patterns measured in this study for volume, diameter and other parameters were transferred under a gap of 540 μm. To ensure sufficient light exposure for high-speed imaging, the gap was increased to 1080 μm.

### 2.3. Characterization Method

**Figure 5:**
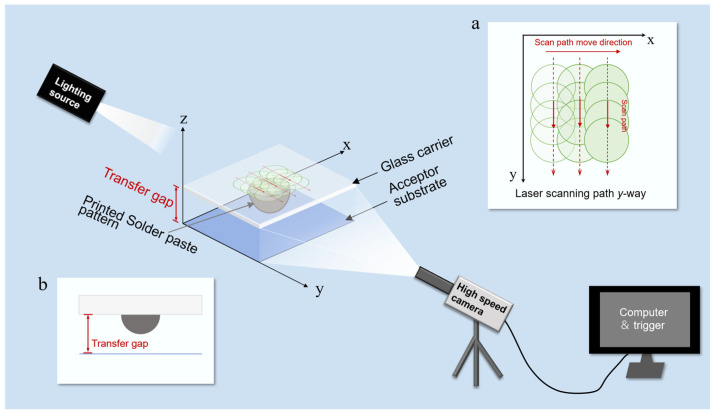
High-speed photography setup. (**a**) Schematic of the path of the galvanometer (*Y*-axis perpendicular to the high-speed camera). (**b**) Schematic of the image captured by the high-speed camera.

The high-speed photography equipment, which consists of high-speed cameras (I-SPEED 221, iX Cameras, Rochford, UK), a magnifying lens (Navitar 12X Zoom, Navitar, Rochester, NY, USA), an illumination source (525 nm MODEL U-40S, DannyU, Shanghai, China), a synchronous trigger, and a computer control system, is shown in Figure 5. The magnifying lens was mounted at the front end of the high-speed camera with a working distance ranging from 15 to 80 mm, and the camera was positioned directly in front of the transfer gap, focusing on the interface between the glass carrier and the solder paste. The illumination source was placed on the opposite side of the camera to form a straight line with the solder paste pattern and provided illumination via backlighting. The morphology of the printed solder paste pattern in spherical cap (PSP) and the transferred solder paste deposit in spherical (TSD) was characterized using optical microscopy (OM, MD-50, UM200i, Mshot, Guangzhou, China) and laser scanning confocal microscopy (LSCM, OLYMPUS LEXT OLS5100, Olympus, Tokyo, Japan).

## 3. Results and Discussion

### 3.1. Description and Analysis of the Transfer Process

**Figure 6:**
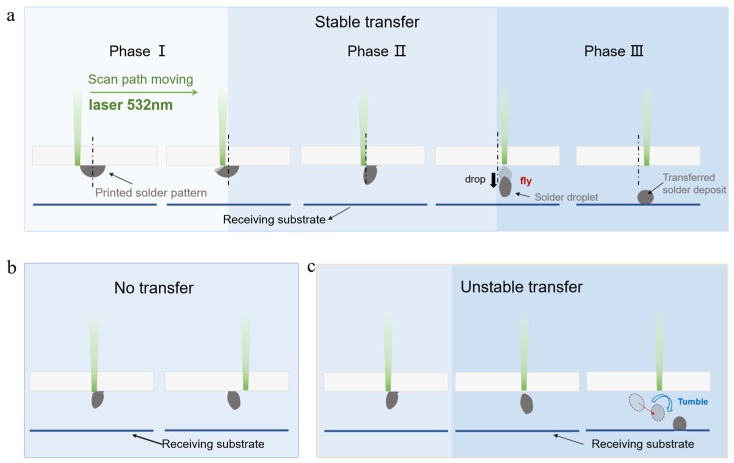
(**a**) Schematic diagram of the phases of stable transfer. (**b**) Schematic diagram of the no transfer. (**c**) Schematic diagram of the unstable transfer.

A high-speed camera system was used to observe and categorize the transfer behavior of solder paste. The frame rate and exposure time were 5000 fps and 200 μs, respectively. The transfer behavior of the solder paste can be categorized into three distinct regimes: stable transfer, unstable transfer, and no transfer, as illustrated in Figure 6. The stable transfer process can be divided into three sequential kinetic phases based on the morphology of the solder paste and the accumulation of kinetic energy: (I) front-edge penetration, (II) gap expansion–viscoelastic tearing, and (III) separation followed by inertial flight. The aspect ratio (AR) is defined as the height of a feature divided by its base diameter: *AR* = *H*/*D*, where *H* is the vertical height and *D* the diameter at the substrate level. This definition is applied consistently to the stencil aperture, the PSP and the TSD.

#### 3.1.1. Front-Edge Penetration

**Figure 7:**
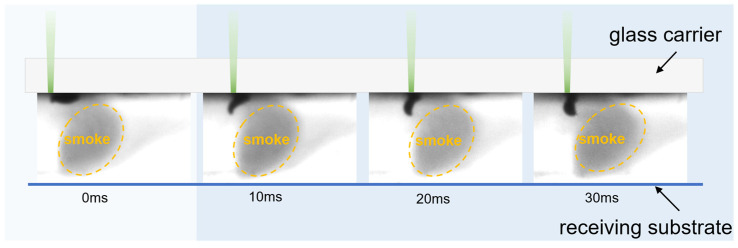
Smoke in high-speed photographic images is capable of stable transfer.

The initial laser pulse was applied to the leftmost edge of the solder paste and propagated toward the right. At this phase, the solder paste pattern remained largely stationary as a whole, with only the leading edge exhibiting micrometer-scale elevation. The separation of the solder paste from the interface with the glass carrier was driven by a dual mechanism involving thermally induced stress and pressure rebound. First, due to the difference in CTE (Coefficient of Thermal Expansion) between the solder paste and glass, thermal mismatch stress is generated after the laser heat source is applied [41]. For this analysis, it is assumed that the laser absorption efficiency of the solder paste and the resulting heat distribution remain constant during the scanning process, ensuring a consistent thermo-mechanical response. The thermo-mechanical stress is given by Equation (1).

(1) $$\sigma_{thermo}=E_{eff}\;(\alpha_{solder}-\alpha_{glass}\;)\;\Delta T/(1-\nu )\;\;$$

where $E_{eff}$ is the equivalent interface modulus, $\alpha_{solder}$ and $\alpha_{glass}$ are the coefficients of thermal expansion (CTE) of the solder paste and glass carrier, respectively; ν is Poisson’s ratio of the solder paste; and ΔT represents the laser-induced temperature rise. Meanwhile, a distinct endothermic peak at 218.4 °C is shown in the differential scanning calorimetry (DSC) curve in Figure 3d, which is consistent with the melting phase transition behavior of the SAC305 (Sn96.5/Ag3.0/Cu0.5) alloy in the solder paste. A 4.2% mass loss in the solder paste between 25 °C and 218.8 °C is revealed by thermogravimetric analysis (TGA). Before the metal alloy melts, solder pastes with lower viscosity show higher organic evaporation rates than those with higher viscosity, indicating localized vaporization of low-boiling-point solvent [42]. At low temperatures, most solder paste remains solid, confining vapor phase products in interfacial microcavities and pores to generate localized vapor pressure $\sigma_{vap}$, which triggers flux components’ phase explosion. As shown in Figure 7, the use of back-illumination reveals a dark shadow moving from left to right along the laser trajectory, which represents clusters of solid particles formed by the cooling of volatilized organic matter within the transfer gap.

Concurrently, the temperature increase reduces the viscosity of the solder paste, and applying increasing shear forces further accelerates this trend. The combined effect of these two mechanisms significantly improves the flowability of the solder paste. The vapor pressure, in conjunction with thermal mismatch stress, overcomes the adhesive strength ($\sigma_{adh}$) at the interface and can be expressed as Equation (2):

(2) $$\sigma_{thermo}+\sigma_{vap}>\sigma_{adh}$$

**Figure 8:**
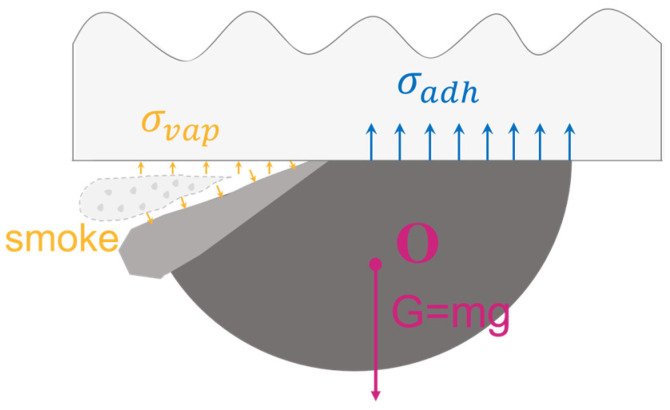
Schematic of forces acting on the solder paste pattern during interface separation.

This breaks interfacial chemical bonds, including van der Waals forces and metal-oxide coordination bonds, and ultimately induces the formation of an initial separation gap [43]. The forces acting on the solder pattern, including gravitational force G, adhesive forces $\sigma_{adh}$ at the glass interface, and counteracting air pressure forces $\sigma_{vap}$, among others, as illustrated in Figure 8.

#### 3.1.2. Interfacial Separation During Gap Expansion

Subsequent to the initial phase dominated by vaporization-induced phase transitions, the laser continues its emission along the predetermined path in a line-scan mode. During this phase, the periodic energy input leads to a continuous expansion of the interface gap.

In this phase, the contour and center of mass of the solder paste are defined as follows: the center of mass is denoted as point O; the leftmost contact point between the solder paste and the glass carrier is designated as point O′; the outermost point of the detached region of the solder paste is defined as point A; the single solder paste pattern is divided into two distinct areas, including the bridged section and the cantilevered section, and the distinct boundary between the interface bridged and the cantilevered section is identified as point B. It was revealed by high-speed photography results that after each 7.8 ms laser path scan (with a 50 μm hatch overlay), the point O′ of the solder paste advanced by approximately 30 to 40 μm.

**Figure 9:**
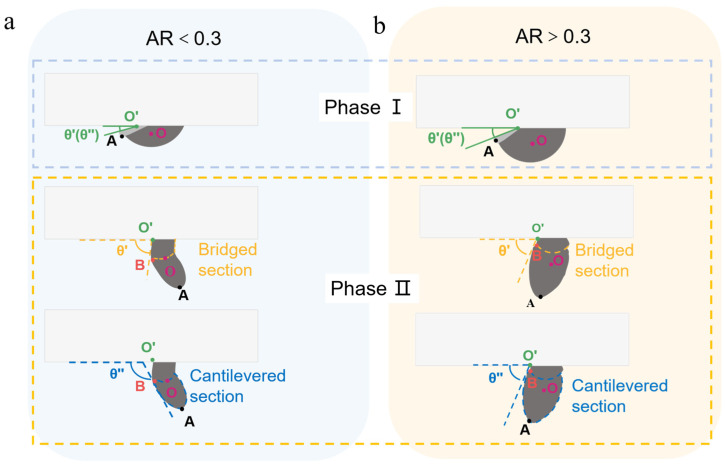
Representative morphologies of solder paste patterns during transfer, highlighting the formation of (**a**) bridging and cantilevered sections at AR < 0.3 and (**b**) a unified structure at AR > 0.3.

As shown in Figure 9, the angles θ′ and θ″ were defined relative to the left side of the glass carrier: θ′ is the angle formed with the tangent drawn from point O′ to the bridged interface, while θ″ is the angle formed with the tangent to the cantilevered section of the signal solder paste pattern.

The volume of the cantilevered section gradually expanded as the laser path was scanned. It was revealed by high-speed photography that after the pattern’s center of mass was scanned by the laser, the cantilevered section underwent a rapid and significant angular rotation, and the bridged section remained constrained by interfacial adhesion; its angle (θ′) only gradually increased under the sustained action of subsequent laser pulses until fracture and detachment occurred.

**Figure 10:**
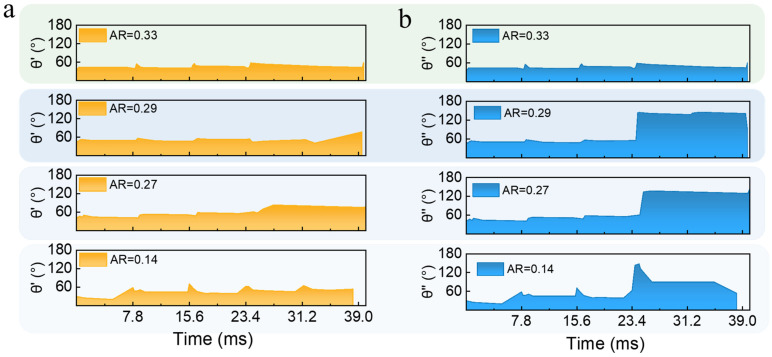
Comparison of angular evolution for 400 μm diameter patterns with varying aspect ratios (AR). (**a**) Bridged section angle (θ′) vs. time. (**b**) Cantilevered section angle (θ″) vs. time.

Notably, solder paste patterns with different aspect ratios (ARs) exhibited distinct mechanical behaviors during the second phase. The angular variation in θ′ and θ″ for solder paste patterns with different aspect ratios (ARs) but a uniform diameter of 400 μm is compared and illustrated in Figure 9, which reveals a critical transition in mechanical behavior. The distinct dynamics of these two regimes are detailed in the high-speed photography sequences presented in Figure 10. As shown in Figure 10a, the low AR of PSP (AR < 0.3) exhibited a pronounced separation phenomenon. In contrast, the bridged and cantilevered sections behaved cohesively for a high-AR of PSP (AR = 0.33). As shown in Figure 10b, the angles θ′ and θ″ remained closely aligned throughout the transfer, and the entire solder pattern detached cleanly from the carrier at a constant angle of approximately 70–90°. Furthermore, Table 2 and Table 3 detail the angular values and corresponding high-speed photography images for patterns with a 400 μm base diameter at AR = 0.14 and AR = 0.33, respectively.

This phenomenon could be attributed to the fundamental geometric differences between solder pastes with varying ARs. A smaller AR means the height and volume of the solder paste pattern to be transferred are reduced. The detached left part of the solder paste with a low aspect ratio acquired greater kinetic energy when exposed to a laser beam with the same trajectory and energy density, which in turn caused it to oscillate more vigorously.

To provide a mechanistic explanation for these AR-dependent dynamics, a first-principles analysis of the competing physical forces is required. The transfer process is governed by a complex interplay between the laser-imparted inertial force, the material’s internal viscoelastic response, and the cohesive force of surface tension. The relative dominance of these effects can be compared using dimensionless numbers [18].

1. Weber number

The Weber number (*We*) is a dimensionless parameter in fluid mechanics used to characterize the relative importance of a fluid’s inertia compared to its surface tension. It is defined by Equation (3).

(3) $$We=\rho v^{2}L/\sigma$$

where ρ is the fluid density, v is the characteristic velocity, *L* is the characteristic length scale (the diameter of PSP), and *σ* is the surface tension of the solder paste. The properties of the SAC305 series solder paste (density ρ  ≈ 7.37 g/cm^3^, viscosity μ ≈ 110 Pa·s), typical LIFT parameters (characteristic length L ≈ 400 μm, velocity v ≈ 0.2 m/s), and a representative surface tension for the organic flux content (σ  ≈ 0.03 N/m) were used. The Weber number (*We*) of approximately 4 was calculated for this process, indicating that the laser-imparted inertial forces were sufficient to overcome surface tension and initiate the significant rotation of the cantilevered section.

2. Deborah’s number

The Deborah number compares the material’s characteristic relaxation time to the process timescale, indicating whether it behaves more like an elastic solid (*De* ≫ 1) or a viscous fluid (*De* ≪ 1). It is defined by Equation (4):

(4) $$De=\lambda /t_{p}$$

where λ is the intrinsic relaxation time of the material and $t_{p}$ is the characteristic timescale of the deformation process.

Solder paste exhibits viscoelastic behavior governed by both elastic effects, characterized by the storage modulus G′, and viscous effects, characterized by the loss modulus G″ [19,44]. As shown in Figure 3b, it was found from Small-Amplitude Oscillatory Shear (SAOS) frequency sweep tests that G′ and G″ intersect at a critical frequency: ${\;f}_{c}=100\;\mathrm{Hz}$. The characteristic relaxation time of the solder paste used in this work was $\lambda =1/\omega_{c}\;=1/(2\pi f_{c}\;)\approx 1.59\;\mathrm{ms}$, reflecting the time scale over which the material responds to deformation.:

- The laser pulse width is 12 × 10^−12^ s, yielding a Deborah number ${De}_{1}=\frac{\lambda }{t_{pulse}}\approx 1.45\times 10^{8}$, significantly greater than 1, which indicates that the material behaves elastically at the single-pulse time scale;
- During laser scanning across the solder paste pattern, the path duration ranges from 10 to 33 μs, corresponding to ${De}_{2}=\frac{\lambda }{t_{path}}\gg 1$, further confirming elastic solid-like behavior and the capacity for elastic energy storage;
- The interval between adjacent laser paths is 7.8 ms, resulting in ${De}_{3}=\frac{\lambda }{t_{interval}}\approx 0.20<1$. This extended interval represents a force-free period during which viscoelastic recovery occurs. It was clearly shown by the analysis of the high-speed image sequences that “step-rebound” angular fluctuations exist, which directly indicate the presence of viscoelastic relaxation. The measured relaxation time ($t_{relax}$) ranged from 0.2 to 3.6 ms during the inter-path gap, allowing sufficient time for material relaxation and resulting in viscous flow behavior.

3. Capillary number

The Capillary number (*Ca*) is used to characterize the competition between viscous forces and surface tension. It is defined by Equation (5):

(5) Ca=  μv⁄σ

where *μ* is the viscosity, *v* is the characteristic velocity, and *σ* is the surface tension. For this process, using representative values of *μ* ≈ 110 Pa·s, *v* ≈ 0.1 m/s, and *σ* ≈ 0.03 N/m, a Capillary number of *Ca* ≈ 366 was calculated. As *Ca* ≫ 1, this indicates that viscous forces are dominant over surface tension, and the role of the latter in maintaining the deposit’s morphology is secondary.

The low-AR patterns exhibited a response characterized by flexible-body dynamics, which was initiated by the high initial velocity imparted to the deposit due to its smaller mass. At such a high rate of deformation, the material’s viscoelastic nature, which had been well established in prior rheological studies of solder pastes, became the critical factor in its response. A response analogous to that of a flexible beam was induced in the low-AR geometry by the combination of the high-velocity impulse and a high Deborah number. This resulted in the large, elastic angular rotation and vigorous oscillation that were observed in Figure 9a. The material’s cohesion was overcome by the laser-imparted inertial force—which, as is indicated by the Weber number, was of a comparable magnitude to surface tension—resulting in the pronounced separation phenomenon.

In contrast, a response analogous to that of a rigid body was observed for the high-AR patterns. This behavior is attributed to two primary factors. First, the larger mass of the high-AR pattern resulted in a lower initial velocity upon laser impact. Second, its slender geometry possesses a significantly larger moment of inertia (I), which for a cylinder rotating about its base is approximated by Equation (6).

(6) $$I=\frac{1}{3}mH^{2}$$

where m is mass and H is the height. According to the principles of rotational dynamics, as described by the equation τ = Iα, a greater moment of inertia imparts a higher resistance to the angular acceleration (α) induced by a given torque (τ) from the laser pulse.

#### 3.1.3. Separation Followed by Inertial Flight

**Figure 11:**
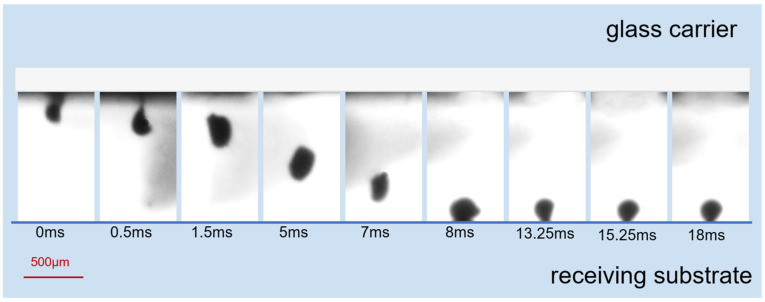
Time-sequence images of solder paste pattern evolution in the third phase.

The final laser loading caused the residual bridged section to completely separate from the glass carrier, allowing the droplet of solder paste to enter the 1080 μm gap in a flying manner for free flight. The evolution of a single solder paste pattern (a diameter of 400 μm, height of 130 μm) during the third phase is demonstrated in the high-speed photography sequence in Figure 11. The bridged section of the solder paste pattern detached from the glass carrier.

A significant morphological transformation is observed throughout the process, becoming particularly pronounced as the angle with the donor carrier passes through the 70–90° range. It gains a certain velocity and falls within the gap along this velocity direction in this shape. During flight within the transfer gap, the solder paste elongated under gravitational force, increasing the ratio of its vertical to horizontal dimensions and adopting a more slender shape. As shown in Figure 8, the vertical velocity of the solder paste pattern is quantified at approximately 0.1 m/s. The solder paste continues its flight within the transfer gap before impacting the receiving board. Upon collision, the vertical dimension of the solder paste contracts. Subsequently, under the influence of surface tension, it contracts into a spherical shape. The duration from contact with the receiving board to spherical contraction is 2 ms.

### 3.2. Changes in Solder Paste Pattern Morphology During the Transfer Process

Statistical comparisons were conducted on the aperture size, including the diameter, height, and aspect ratio of the stencil aperture used in the experiment, the single printed solder paste pattern, and the transferred solder paste deposit. Due to the stencil design requirement that the aspect ratio (aperture diameter/stencil thickness) ≥ 2.5, the ARs of PSP were approximately 0.2–0.4.

**Figure 12:**
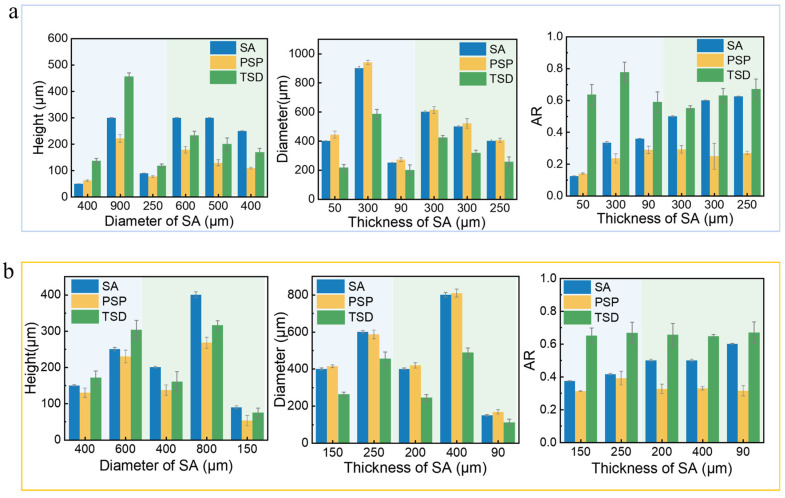
(**a**) Size comparison of SA and TSD for PSP with AR < 0.3. (**b**) Size comparison of SA and TSD for PSP with AR > 0.3.

As seen in Figure 12, the diameter of the printed solder paste pattern is slightly larger than the stencil aperture diameter, indicating no spread phenomenon. The aspect ratio (AR) of the printed solder paste pattern was classified into three distinct intervals: AR > 0.3, 0.15 < AR < 0.3, and AR < 0.15.

1. When the AR of PSP > 0.3, the transferred solder paste deposit exhibits a stabilized aspect ratio of approximately 0.7, doubling the initial value of PSP. The height increases to 1.27 times the PSP value, while the diameter decreases to about 0.66 times the original.
2. For the intermediate range (0.15 < AR of PSP < 0.3), the aspect ratios of the transferred solder paste deposits range between 0.6 and 0.8, also increasing to over twice the pre-transfer value. The height rises to about 1.6 times, and the diameter contracts to 0.66 times the pre-transfer measurements.
3. When the AR of PSP < 0.15, the aspect ratios of the transferred solder blob similarly fall between 0.6 and 0.8. Due to the significantly larger pre-transfer diameter relative to height, rotational effects during transfer cause the aspect ratio to increase by more than fivefold. The height increases to approximately 2.2 times, and the diameter decreases to about 0.49 times the pre-transfer values.

Under the influence of surface tension, which manifests as the Plateau–Rayleigh instability [45], the process resulted in the formation of an approximately spherical solder paste deposit at the receiving substrate, with no observed breakage or satellite splatter. During the first phase and secondary phase of the transfer process, the solder paste underwent a rotation of approximately 70~90° before arriving on the receiving substrate. This rotation transformed their morphology from a low aspect ratio configuration to one in which the vertical dimension exceeds the horizontal dimension. Such structural reorientation facilitated an increased aspect ratio during subsequent reshaping after landing, representing a critical factor in the transfer mechanism. As shown in the high-speed photography sequence of Figure 11, the flight of a solder droplet through the transfer gap and its subsequent deposition onto the receiving substrate were recorded.

**Figure 13:**
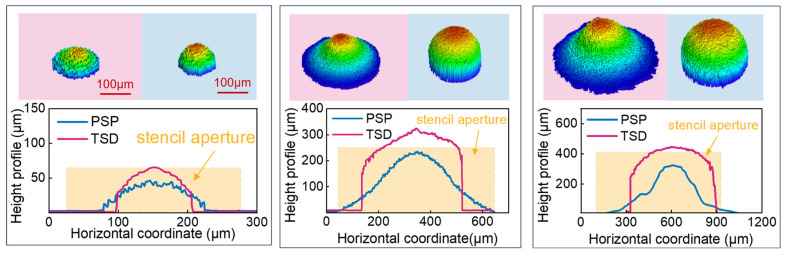
Comparison of contours for three types of solder dots before and after transfer.

It is revealed in Figure 13 that TSDs possess smaller diameters and higher aspect ratios than the PSPs. It should be noted, however, that the volume of these spherical deposits is slightly overestimated by confocal microscopy, as its topography-based calculation misinterprets the shadowed undercuts as solid material. To account for this measurement artifact, the raw measured volume values of transferred solder paste deposits can be corrected to account for measurement artifacts. After correction, these values were found to correlate well with the initial volume of the printed solder paste pattern.

**Figure A1:**
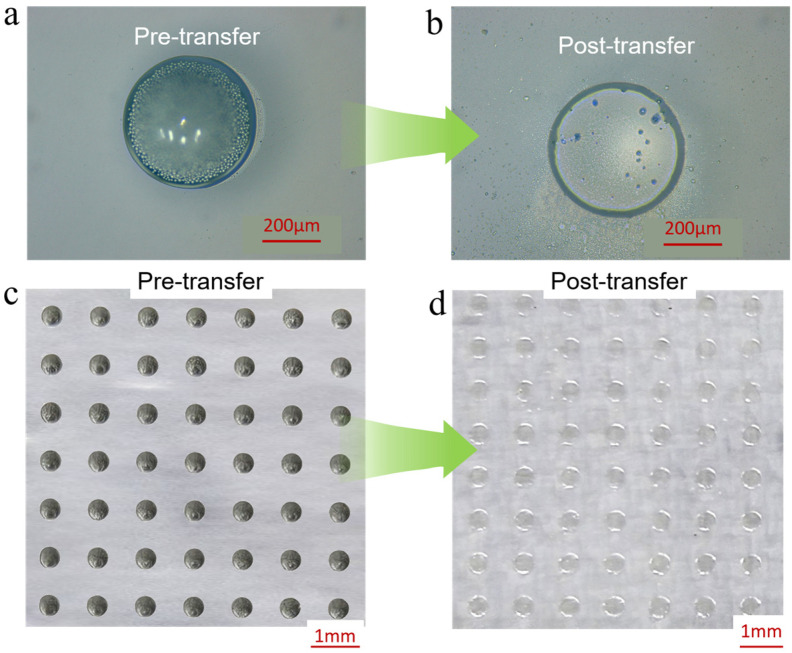
Optical comparison of the glass carrier before and after laser transfer. (**a**) Optical micrograph of the solder paste pattern on the glass carrier before transfer, focused on the pattern’s edge (the central area appears defocused due to the pattern height). (**b**) Optical micrograph of the same glass carrier after transfer, showing negligible residue. (**c**) Macroscopic photographs of the glass carrier with the full solder paste array before transfer and (**d**) after the array has been transferred.

The dimensional characteristics of the printed solder paste pattern are significantly affected by the dimensions of the stencil aperture in relation to the design standards for solder placement. In case the stencil fails to meet this standard, solder paste patterns with heights ranging from 43% to 67% of the stencil thickness are produced, accompanied by substantial volume loss, as shown in Figure 4(aiii). Furthermore, solder paste trapped in the stencil apertures after printing may lead to issues such as stencil corrosion, clogging, and difficulties in cleaning [46]. As shown in Figure A1, this method achieved virtually no residue on the carrier after transfer, with only a few solder beads ranging from 5 to 15 μm in size, indicating negligible solder paste loss. The volume loss from the process of transfer can be attributed to the presence of solder paste residue on the aperture walls during the process of lifting the stencil.

The quantitative analysis of alterations in solder paste dimensions during the scraping and transfer phases provided a foundation for the evaluation and reference of this technique in production settings.

### 3.3. Energy Threshold Transferable by Solder Paste Globules

#### 3.3.1. Effect of Laser Fluence on the Transfer Process

Laser fluence (F) is the key control parameter determining whether solder paste can be successfully peeled off and transferred. This experiment uses picosecond pulses with a pulse width of T = 12 ps and a wavelength of λ = 532 nm. Figure 2b shows the energy distribution along the emission trajectory, where the effective region can be approximated as a plane with uniform energy density. Statistical results indicate that the volume (V) of transferable solder paste pattern exhibits a monotonically increasing relationship with F, and a distinct energy-window transferability phenomenon is observed: for any given initial volume V_0_ of Type 6 sprayed solder paste, there corresponds a stable transfer interval defined by the lower peel threshold fluence $F_{C,lower}$, and the upper damage threshold fluence $F_{C,upper}$. This interval can be quantitatively described as:

(7) $$F_{C,lower}<F<F_{C,upper}$$

When F < $F_{C,lower}$, the peak vapor backpressure $\sigma_{vap}$ in the interface is less than $\sigma_{adh}$, preventing the two from separating due to cracking. As shown in Figure 6c, the solder paste remains in a viscoelastic state and adheres to the substrate, with no mass loss. When F > $F_{C,upper}$, the upper instantaneous temperature rise triggers flash pyrolysis and micro-explosive degassing of the organic carrier in the solder paste. As shown in Figure 7, high-speed photography captured the violent vapor plume condensing into solid particles upon cooling, confirming the micro-explosive boiling of organic materials during the first phase of separation between the solder paste pattern and glass carrier interface, generating a pressure rebound force. Visible black marks on the glass carrier indicated severe damage due to excessive energy input, including intense oxidation of organic materials.

As demonstrated in Figure 6c, a higher initial velocity is attained by the solder paste pattern due to excessive energy input within the same time period. The system’s short rebound time results in inadequate rebound, leading to a shift in the center of mass to the right. When subjected to the final pulse column, the solder paste deflects to the right during flight and undergoes rapid rotation within the gap. The angular velocity of the object is measured at 60,000 rad/s. This phenomenon led to substantial positional deviation and unstable transfer, attributable to the volatilization and oxidation of components. In addition, the multiple rolling trajectories of the solder droplet along the glass substrate caused lateral sliding and overturning torque during the post-detachment flight phase, resulting in a landing point error, failing to meet the packaging precision requirement.

#### 3.3.2. Energy for Stable Transfer

The transfer behavior of printed solder paste patterns is found to be strongly influenced by their geometric shape. Geometric variations in stencil-printed solder deposits, resulting from process deviations, necessitate systematic energy threshold analysis against key dimensional parameters. Transfer energy is correlated with paste height and base diameter, providing direct guidance for industrial process optimization.

**Figure 14:**
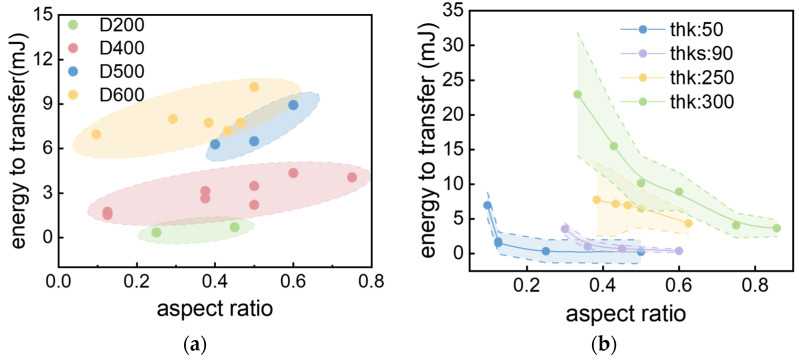
Total energy required for transfer versus solder paste deposits. (**a**) Energy response of aspect ratio distribution under different aperture diameters of stencil aperture, (**b**) Energy response of aspect ratio under different thicknesses of stencil aperture (in the diagram, “thk” is an abbreviation for “thickness,” referring to the stencil’s thickness, which can also be understood as the aperture depth of the steel mesh. “D” denotes the diameter of the steel mesh aperture).

The established relationships assist in selecting stencil parameters and laser energy windows, reducing trial-and-error efforts while offering insights into microscale laser material interactions. As illustrated in Figure 14, for a fixed diameter, the required laser energy density E is observed to increase monotonically with pattern height. Conversely, for a fixed height, E decreases monotonically as the aspect ratio of PSP increases. These combined trends suggest a positive correlation between the transfer energy threshold and the volume of the solder paste pattern, which can be expressed by the relationship $E\propto V^{\alpha }$. The morphology of the solder paste pattern after stencil printing can be regarded as a spherical crown, whose volume is expressed in Equation (8):

*V *=* πh*/6 · (*3a*^2^*+ h*^2^) (8)

where *a* is the radius of the base, and *h* is the height of the cap. The experimental energy threshold E and *V* satisfy the condition shown in Equation (9).

(9) $$E=k\cdot V+\gamma_{int\;}\;\cdot \pi a^{2}$$

The first term *k*·*V* represents the energy requirement for the laser to act on the entire pattern until the interface peels off and imparts kinetic energy to it. The second term $\gamma_{int\;}\;\cdot \pi a^{2}$ is the fixed energy consumption required to overcome the adhesive work at the interface between the glass carrier and solder paste pattern, including thermal stress and the energy from the backpressure of organic compounds. $\gamma_{int\;}\;$ is the interfacial specific energy.

During transfer, if the material fails to separate completely from the glass substrate, the critical separation point reaches the elastic stored energy E of the solder paste pattern when subjected to laser pulses. Within the laser pulse scanning interval, the release of this stored energy E causes a portion of the solder paste at the critical separation point to reattach to the glass substrate. Consequently, solder paste patterns with lower height require multiple scans to ensure the reattached portion is completely separated from the glass substrate and successfully transferred.

As shown in Equation (9), when h is constant and a increases, *V* is proportional to *a*^2^, while the interface term: $\gamma_{int\;}\;\cdot \pi a^{2}$ also increases linearly with *a*^2^. This result is consistent with the macroscopic peeling experiment, which shows that the adhesion peeling energy increases linearly with the contact area.

For a constant diameter a, the volume V as a function of height h is given by the approximation in Equation (10), where the term $O(h^{3})$ becomes negligible for small *h*.

(10) $$V\;\approx \;\pi h/6\;\cdot \;(3a^{2}+h^{2})\;\approx \;\pi a^{2}h/2+O(h^{3})$$

For h ≪ a, the volume scales linearly with height (V ∝ h), as the *h*^3^ term is insignificant, and the interfacial energy term $\gamma_{int}\cdot {\pi a}^{2}$ is independent of *h*. The resulting dependence of E on h is therefore sublinear, tending toward a plateau for large *a*, which explains the experimental finding that the energy threshold remains largely constant with increasing height. This phenomenon can be reasonably explained from two perspectives: “interface–volume coupling” and “thermal–mechanical energy coupling”. This explains the macroscopic phenomenon observed in experiments where “increasing diameter at the same thickness leads to a sharp rise in energy, while increasing thickness at the same diameter results in a gradual increase in energy,” providing a rigorous mathematical boundary for the precise design of the laser-induced transfer process window.

#### 3.3.3. Relationship Between Solder Volume and the Viable Energy Density Window

**Figure 15:**
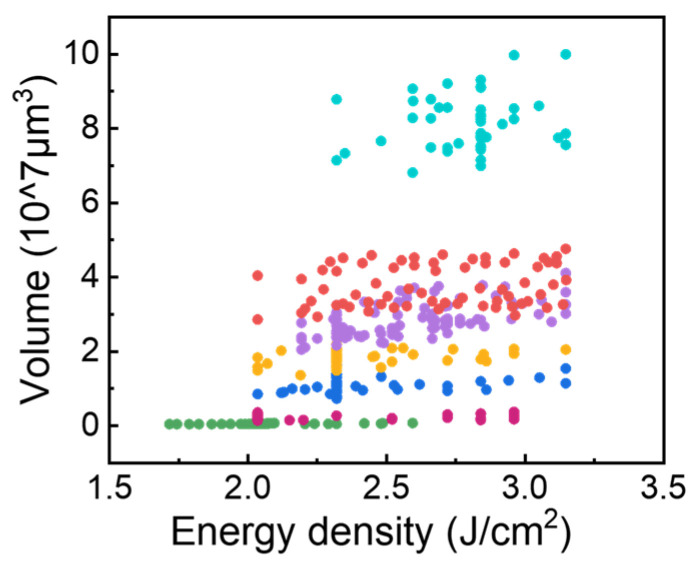
Volumetric distribution of solder paste deposits at various transfer energy densities (each color group corresponds to a solder paste deposit printed with the same stencil aperture size and has a similar initial volume).

While the total transferred energy—the product of single-pulse energy and pulse count—governs the transfer of a uniform solder pattern, it is an insufficient metric for processes involving variable-sized material deposits. For such multi-size applications, energy density is the more fundamental and robust control parameter; it is directly governed by set-point parameters like laser power and scan strategy, making it inherently independent of the target material’s dimensions. In this context, we distinguish total energy F, from energy density, denoted by f. While F varies with deposit size, f is an intrinsic process parameter. Furthermore, Figure 15 shows the energy density window map of the solder paste pattern with different volumes $V_{0}$. It can be observed that:

As V_0_ increases, both $f_{c,\;lower}$ and $f_{c,\;upper}$ shifts toward the high-energy side, the interval width ${\Delta f=f}_{c,\;lower}-f_{c,\;upper}$ is distributed between 0.937~1.112 J/cm^2^, consistent with the volume-energy coupling saturation effect. Additionally, the laser power used in the experiment has reached its upper limit, making it impossible to increase the power to verify the transfer upper limit for a larger-volume solder paste pattern.

In summary, the process achieves stable, morphology-preserving transfer of solder paste patterns. The reported 0.937–1.112 J/cm^2^ energy density window highlights significant process redundancy, as visualized in Figure 15. This redundancy manifests in two key ways: (1) patterns of a similar volume demonstrate transfer stability across the entire window, and (2) a single energy density (e.g., 2.5 J/cm^2^) can successfully transfer a wide range of different volumes. This behavior is a significant advantage over conventional LIFT processes, which are typically highly energy-sensitive and prone to deposit size variations; the hybrid method thus ensures superior transfer stability [26,28].

## 4. Conclusions

This work presents an innovative integration of stencil printing and LIFT that circumvents the key drawbacks of each technique. This hybrid technique transfers high-viscosity (110 Pa·s) paste for high-aspect-ratio deposits without the splashing seen in conventional LIFT. Some conclusions can be highlighted as follows:

- The transfer mechanism is governed by laser-induced vaporization pressure, which initiates separation at the solder–glass interface. This separation is then driven progressively along the scan direction by a “micro-chiseling effect,” created by the periodic pressure gradient (∇P) from successive laser paths. The material’s viscoelasticity governs the dynamic response to these forces, as evidenced by the partial angular rebound observed during this tearing process.
- The transfer dynamics were found to be critically dependent on the initial aspect ratio (AR). Low-AR patterns exhibited a distinct bifurcation into bridging and cantilevered sections, whereas high-AR patterns demonstrated a more cohesive, rigid-body-like detachment.
- Compared to the printed solder paste (PSP) patterns, the transferred solder paste deposits (TSDs) exhibited substantially higher aspect ratios, reaching approximately 0.7. Analysis across different solder volumes revealed a positive correlation between the required transfer energy and the solder volume.
- The window’s boundaries were defined by incomplete detachment ($F<F_{C,lower}$) and unstable transfer ($F>F_{C,upper}$). The process demonstrates significant process redundancy (Figure 15) within an established stable transfer window of 0.937–1.112 J/cm^2^. This redundancy—where a wide range of volumes can be transferred at a single energy density, or a single volume is stable across the entire window—ensures superior morphology preservation.

This method’s demonstrated large-gap, non-contact transfer process establishes a solid foundation for advanced manufacturing, enabling 3D integration that surpasses the geometric constraints of stencil printing. This capability opens up key application frontiers, particularly in the conformal patterning of System-in-Package (SiP) modules and high-power devices that benefit from high-aspect-ratio interconnects.

## Figures and Tables

**Table 1:** Properties of solder paste.

M705-LFAC60-Type6D-15.0					
Type	Particle Size	Components	Organic Solvent Content	Viscosity	Melting Point
Type6	5~15 μm	Sn96.5/Ag3.0/Cu0.5	15 ± 1%	110 ± 20 Pa·s	217~220 °C

**Table 2:** θ′ and θ″ vs. time of a printed solder pattern with AR = 0.14.

Time(ms)	7.8	9.8	15.6	19.0	24.2	26.2
θ′ (°)	58.6	45.0	70.9	41.2	50.5	48.4
θ″ (°)	58.6	45.0	70.9	41.2	137.9	95.1
image	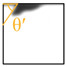	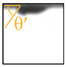	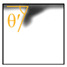	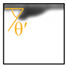	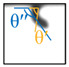	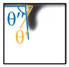

**Table 3:** θ′ and θ″ vs. time of a printed solder pattern with AR = 0.33.

Time(ms)	7.8	15.6	15.8	23.4	33.4	39.0
θ′ (°)	39.1	41.4	51.3	40.4	46.4	37.2
θ″ (°)	39.1	41.4	51.3	40.4	50.6	43.5
image	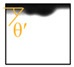	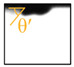	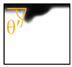	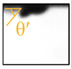	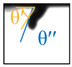	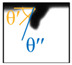

## Data Availability

The original contributions presented in this study are included in the article. Further inquiries can be directed to the corresponding authors.

## Abbreviations

The following abbreviations are used in this manuscript:

LIFT	Laser-induced forward transfer
BGA	Ball grid array
AR	Aspect ratio
SAOS	Small-Amplitude Oscillatory Shear
PSP	Printed solder paste pattern
TSD	Transferred solder paste deposit
